# Higher pyruvate levels after Achilles tendon rupture surgery could be used as a prognostic biomarker of an improved patient outcome

**DOI:** 10.1007/s00167-020-06037-x

**Published:** 2020-05-06

**Authors:** Gianluigi Capone, Simon Svedman, Robin Juthberg, Gunnar Edman, Paul W. Ackermann

**Affiliations:** 1 Integrative Orthopedic Laboratory, Department of Molecular Medicine and Surgery, Karolinska Institutet, Stockholm, Sweden; 2 Department of Orthopedic Surgery, Karolinska University Hospital, Stockholm, Sweden; 3 Orthopaedic and Traumatology Unit, Ospedale Regionale di Lugano, EOC, Lugano, Switzerland; 4 Faculty of Pharmacy and Medicine, Sapienza University of Rome, Rome, Italy; 5 Department of Psychiatry, Tiohundra AB, Norrtälje, Sweden

**Keywords:** Achilles tendon, Rupture, Biomarker, Healing, Lactate, Pyruvate, Patient-reported outcome measures, Pedometer, Early mobilization

## Abstract

**Purpose:**

The primary aim of this study was to assess the relationship between the metabolites lactate and pyruvate in the healing tendon after Achilles tendon rupture (ATR) and patient-reported outcome at 6 and 12 months. A secondary aim was to evaluate which underlying factors regulate lactate and pyruvate concentrations.

**Methods:**

Lactate and pyruvate concentrations were measured two weeks post-operatively in both the healing- and healthy Achilles tendon in 109 patients (90 men, 19 women; mean age 40 ± 7.9 years). Patient demographics, degree of physical activity, timing of surgery, operation time, patient-reported loading and step counts were investigated in relation to metabolite concentrations. At 6 and 12 months, the Achilles tendon Total Rupture Score (ATRS) questionnaire was used to assess patient outcome.

**Results:**

The mean number of steps taken during the post-operative days 1–10 was the only factor significantly related to the mean concentration of lactate (*R*^2^ = 0.34, *p* = 0.038), and pyruvate (*R*^2^ = 0.46, *p* = 0.006). Pyruvate was demonstrated as the only factor significantly associated with ATRS at both 6 months (*R*^2^ = 0.32, *p* = 0.003) and at 12 months (*R*^2^ = 0.37, *p* = 0.004) using multiple linear regression.

**Conclusion:**

The mean concentration of pyruvate during early ATR healing may predict patient outcome at 6 and 12 months post-operatively and possibly be used as a biomarker of healing. Early mobilization with an increased number of steps taken is an important clinical strategy to improve the metabolite concentrations during healing.

**Level of evidence:**

III

## Introduction

Tendon injuries are among the most common problems in athletes, causing long rehabilitation times and often lead to chronic problems with pain, weakness, impaired function and post-operative complication [1, 13, 18, 24, 26, 27]. Achilles tendon rupture (ATR) has been shown to preclude the return to sports for approximately 20–30% of professional athletes [8]. Among leisure athletes, ATR is associated with unpredictable and often poor long-term patient-reported outcomes [11, 29]. The influence of early mobilization on patient outcome has also been studied during the post-operative rehabiltation after ATR [3, 4, 15]. However, as of today there is no clear understanding of why the outcomes are so poor following ATR. Moreover, there are no biomarkers available that can predict the long-term outcome or be utilized in the development of new targeted therapies following ATR.

Research on the metabolic markers that are essential for repair may give new insight to the tendon healing process. The metabolites lactate and pyruvate, which are formed depending on the availability of oxygen during the glucose metabolism, are vital for energy production that may be essential for healing (Fig. 2). Recently it was demonstrated that alterations of the concentrations of lactate and pyruvate can change the healing progress [31]. More specifically it was shown that downregulation of lactate through inhibition of the enzyme pyruvate dehydrogenase kinase, which increases pyruvate entry into the aerobic metabolism, improved the mechanical properties of healing mouse tendons [31].

Both lactate and pyruvate are key metabolites in the cell energy metabolism and both have been proved to promote wound healing. Lactate has, in cultured tendon cells, been demonstrated to enhance collagen production in vitro. Pyruvate supplementation during wound healing accelerated migration of fibroblasts and myoblasts to promote wound gap closure [16]. Earlier studies have demonstrated that it is possible to assess the concentrations of lactate and pyruvate in human ATR healing using microdialysis [9] and that the ratio of lactate and pyruvate in human ATR healing is related to patient outcome [2]. The ratio of lactate/pyruvate, which is an indirect measure of the concentration of tissue hypoxia, is being used as a clinical marker for patient outcome after traumatic brain injury [11, 19, 22, 23]. However, whether the actual concentrations of lactate and pyruvate are related to patient outcome, and in that case, which underlying factors that may alter these metabolites concentrations during healing, is not fully known.

In this study it was thus hypothesized that the actual concentrations of lactate and pyruvate in the healing after ATR could be used as biomarkers of patient-reported outcome. The primary aim of this study was to assess lactate and pyruvate concentrations in the healing Achilles tendon in relation to patient-reported outcome at 6 and 12 months post-operatively. A second aim was to investigate pre-, per- and post-operative factors that would influence the lactate/pyruvate metabolism and inform about underlying regulatory mechanisms. The findings of this study could potentially lead to new clinically useful treatment strategies involving direct or indirect methods to influence the concentrations of metabolites lactate and pyruvate in the healing tendon, and thus improve patient outcome.

## Materials and methods

Ethical approvals with ID number 2009-2079-31 and 2013-1791-31-3 was obtained from the Regional Ethical Review Committee in Stockholm, Sweden. Between 2010 and 2019, 370 patients with acute ATR were screened for eligibility in three randomized controlled trials that this study was based on [4–6]. The inclusion criteria were acute unilateral ATR, age between 18 and 75 years and reconstructive ATR-surgery performed on the tendon within 7 days from the injury. Exclusion criteria were current anticoagulation therapy, thrombophlebitis, known malignancy, thromboembolic event during the previous 3 months, chronic or acute kidney failure, heart failure with pitting oedema, haemophilia, pregnancy, other surgery during the previous month, lack of ability to follow instructions or planned follow-up at another hospital. In the present study those patients who performed microdialysis were included. Thus, 109 patients were eligible for analysis in this study.

**Fig. 1:**
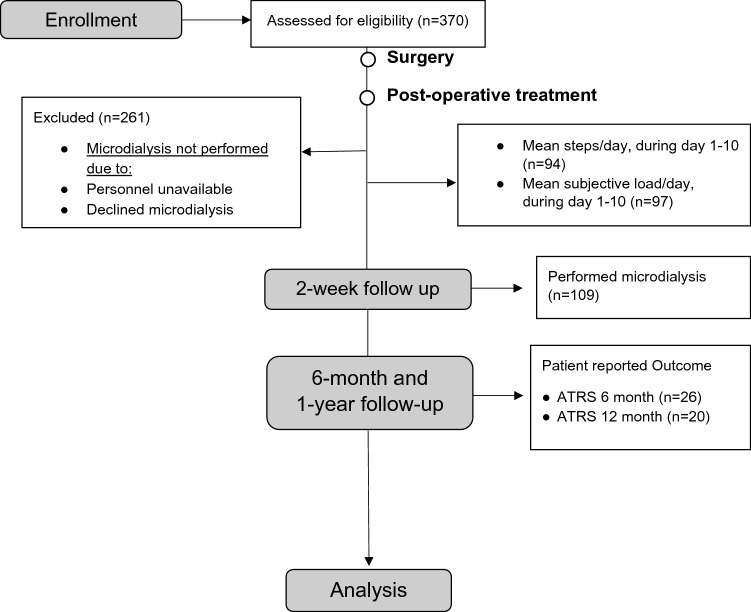
Follow-up flowchart showing patient inclusion and follow-up

All participants received oral and written information about the study protocol and a written informed consent was signed prior to ATR surgery. The patients enrolled in the trials were randomized to the post-operative treatments by a research nurse after surgery (Table 1, Fig. 1).

**Table 1:** Demographic data on study participants

Variable	
Sex (Male/female) *n* (total)	90/19 (109)
Age (years) M (SD)	40 (7.9)
BMI (Kg/m^2^) M (SD)	25.3 (2.7)
Tobacco user no *n* (%)	97 (89.0)
Degree of activity preinjury^a^ M (SD)	4.6 (0.9)
Time from injury to operation M (SD)	79 h (39 h)
Operation time M (SD)	40 min (15 min)
Time from surgery until microdialysis M (SD)	14.9 days (4.1 days)
Mean steps/day, day 1–10 M (SD)	1739.4 (1822)
Mean subjective load, day 1–10 M (SD)	42.2 (21)

*M* mean, *SD *standard deviation, *BMI *body mass index

^a^As estimated on the Physical Activity Scale (PAS)

### Surgical procedure

The surgical procedure, as described earlier [9], was performed on an outpatient basis providing standardized local anesthesia and open surgical techniques, using the modified Kessler suture, according to a predefined study protocol. All ATR surgeries were performed at the same University Hospital.

### Post-operative treatments

Patient included in the original trials underwent different post-operative treatments, which differed mainly during the first two post-operative weeks. Forty-two patients received treatment with plaster cast immobilization [9], 12 patients with calf intermittent pneumatic compression under an orthosis [6], 5 patients with foot intermittent pneumatic compression under plaster cast [9] and 50 patients with functional weight-bearing in an orthosis (VACO^®^ ped, OPED Gmbh, Germany) [3, 25, 27] (Table 2).

**Table 2:** Demographic data in relation to pyruvate/lactate

Dependent outcome variable	Univariate correlations pyruvate mean	Univariate correlations lactate mean
Gender	*r* = 0.08, n.s	*r* = 0.03, n.s
Age (years)	*r* = 0.07, n.s	*r* = 0.09, n.s
BMI	*r* = 0.02, n.s	*r* = − 0.04, n.s
Degree of preinjury activity	*r* = 0.07, n.s	*r* = − 0.02, n.s
Plastercast and foot IPC^a^	*r* = 0.18, n.s	*r* = 0.10, n.s
Calf IPC^a^	*r* = -0.01, n.s	*r* = − 0.12, n.s
Plastercast^a^	*r* = -0.06, n.s	*r* = 0.07, n.s
Vacoped^a^	*r* = -0.02, n.s	*r* = − 0.02, n.s
Time from Injury to surgery	*r* = 0.06, n.s	*r* = − 0.04, n.s
Operation time	*r* = 0.02, n.s	*r* = 0.04, n.s
Time from surgery until microdialysis	*r* = -0.07, n.s	*r* = − 0.11, n.s
Mean steps/day, days 1–10	***r = 0.46, p = 0.006***	***r = 0.34, p = 0.038***
Mean subjective load, day 1–10	*r* = 0.13, n.s	*r* = 0.15, n.s

Bold = significance < 0.05

*n.s *non significant correlation, *IPC *Intermittent pneumatic compression. ^a^post-operative treatments

### Subjective load

From the day after surgery, patients completed a diary on estimated daily weight-bearing load. The percentage of load on the injured side compared to the uninjured side was registered on a scale from 0%-100%. Patient-reported loading has earlier been demonstrated to significantly correlate to plantar flexion force [25].

### Pedometer

The number of steps per day were registered with a pedometer (Yamax SW-200/LS2000; Yamax Corporation), which the patients carried at the hip during the first two weeks following ATR surgery. The Yamax SW-200 has been used as a gold standard pedometer in earlier validation studies and has less than a 3% margin of error and more than 88% of accuracy [21].

### Microdialysis

**Fig. 2:**
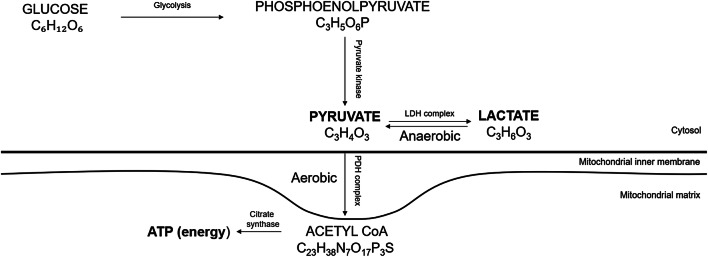
Overview of pyruvate/lactate metabolism

In 109 patients, microdialysis was performed at two weeks after ATR surgery in both the healing and in the contralateral uninjured tendon. A microdialysis catheter (CMA 71; CMA Microdialysis AB, Solna, Sweden; 100 kDa molecular cut-off, 0.5 mm outer diameter; 30 mm in length) was inserted in volar aspect of the paratenon and sampling of healing fluid was continued as earlier described [9]. An examiner blinded to the treatment group performed the microdialysis. Subsequently, the concentration of lactate and pyruvate was quantified using ISCUS Clinical Microdialysis Analyzer. The ISCUS is calibrated using solutions with known lactate and pyruvate concentrations. The assay imprecision is less than 4% relative standard deviation for control samples and the accuracy is more than 90% for controls samples (CMA Microdialysis AB) (Fig. 2).

### Adverse events

An adverse event was noted if the patient exhibited any of the following: DVT as confirmed by Doppler ultrasound at 2 or 6 weeks of follow-up, post-operative infection at 2 or 6 weeks of follow-up, or a rerupture at any time during the year following surgery. The adverse events were reported together as one single factor when correlating to the metabolite concentrations, since the rate of each of the complications described above was low.

### Patient-reported outcome

At six months and again at one year post-operatively the patients completed two different validated (ATRS), Swedish, version 6 (PAS) [18, 20] with the purpose to evaluate the patient’s degree of symptoms.

### Achilles tendon total rupture score (ATRS)

The ATRS includes ten precise queries (scored from 0 to 10, 10 indicating no limitations) regarding pain, fatigue, stiffness and limitations in activities of daily living, hard physical work, walking on uneven surface, sports and jumping [17]. The maximum total score of ATRS is 100, indicating the best possible outcome. Results equivalent to or over 80 in the overall score, and ≥ 8 in the subscales, were considered as “good” subjective outcomes. The ATRS has previously been shown to significantly correlate to the the Disability Rating Index, at five different time points (pre-injury, six weeks, three, six and nine months), while at the same time being better at detecting clinically important changes over time (responsiveness) as compared to both the Disability Rating Index and EQ 5D [12].

#### Physical activity score (PAS)

PAS is a score ranging from 1 to 6, where 1 indicates a sedentary lifestyle for the patient, while a score of 6 indicates that the patient engages in intense physical work several times a week [1, 18].

### Statistical analysis

The sample size was calculated on a minimal expected prognostic correlation coefficient between the concentration of lactate/pyruvate and clinical outcome of ATRS with *r* = 0.30. It was determined that a sample size of 85 patients would be necessary to detect a *r* = 0.30 correlation with 80% power when alpha was set equal to 5%. One-hundred and nine patients in total with lactate/pyruvate assessment were included to account for loss of one-year follow-up data. All data were analyzed using SPSS (IBM SPSS, version 24.0, Armonk, NY, USA). The variables were summarized with standard descriptive statistics such as mean, SD, and frequency. All variables were checked for skewness. Correlations between metabolites and other variables were investigated with Pearson and Spearman correlation for parametric and nonparametric values, respectively. For outcome variables that were normally distributed, multiple linear regression analyses (stepwise forward method with an inclusion concentration of 0.05) were conducted. These analyses were performed to investigate the unique relationship between the independent variables (mean concentration of lactate and pyruvate, age, gender, BMI, pedometer) and the dependent variable. The significance level in all analyses was set at *p* ≤ 0.05 (two-tailed).

## Results

### Lactate and pyruvate concentrations during healing

At two weeks post-injury the mean concentrations of lactate and pyruvate were significantly increased in the healing tendons as compared to the contralateral uninjured Achilles tendons among the 109 included patients. The concentration of lactate was 1.7 ± 0.74 mM (range: 0.33–4.4 mM) in the healing tendon and 0.73 ± 0.32 mM (range: 0.13–1.7 mM) in the contralateral tendon, whereas the concentration of pyruvate was 88.0 ± 26.7 µM (range: 5.33–153 µM) in the healing tendon and 48.2 ± 23.6 µM (range: 4.16–129 µM) in the contralateral tendon.

### Effects of pre- and per-operative factors on lactate/pyruvate concentrations

Neither patient factors (age, gender, BMI), PAS, time from injury to surgery, time from surgery until microdialysis nor the duration of the surgery, were significantly related to the concentrations of lactate or pyruvate that were assessed in the healing tendon at a mean of two weeks after ATR (Table 2).

### Effects of post-operative factors on lactate/pyruvate concentrations

**Fig. 3:**
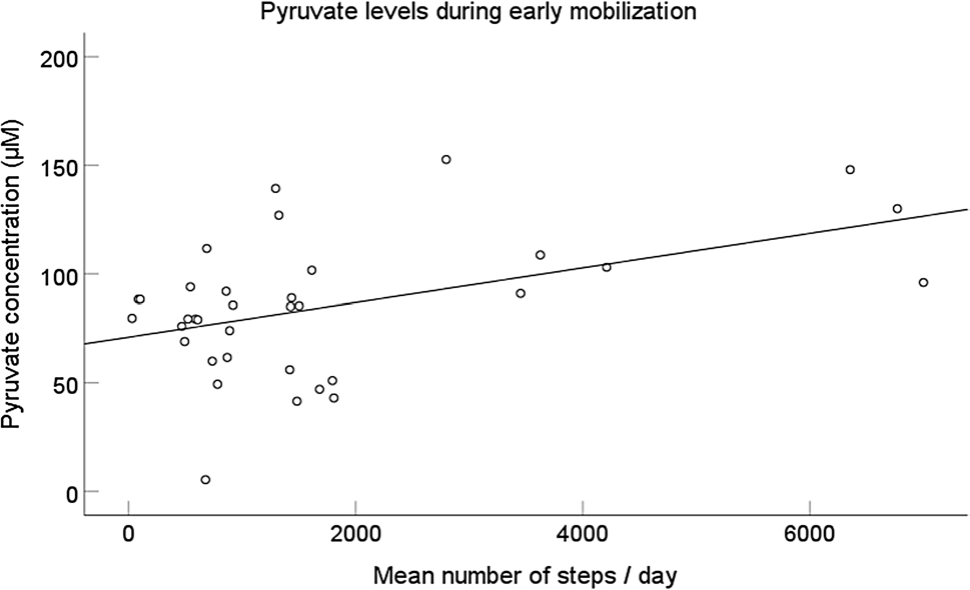
Pyruvate concentration in relation to the mean number of steps taken daily

Higher number of steps taken during the post-operative days 1–10 was correlated to increased concentrations of both lactate and pyruvate in the healing tendon (Table 2, Fig. 3). Multiple linear regressions corroborated a significant correlation between the mean number of steps on the post-operative days 1–10 and the mean concentration of lactate (*R*^2^ = 0.34, *p* = 0.038), and pyruvate (*R*^2^ = 0.46, *p* = 0.006). Neither more weight-bearing, longer time from surgery until lactate and pyruvate assessment nor the type of post-operative treatment, were significantly related to the concentrations of lactate or pyruvate in the healing tendon (Table 2).

### Lactate and pyruvate concentrations related to outcome

#### Outcome at 6 months

**Fig. 4:**
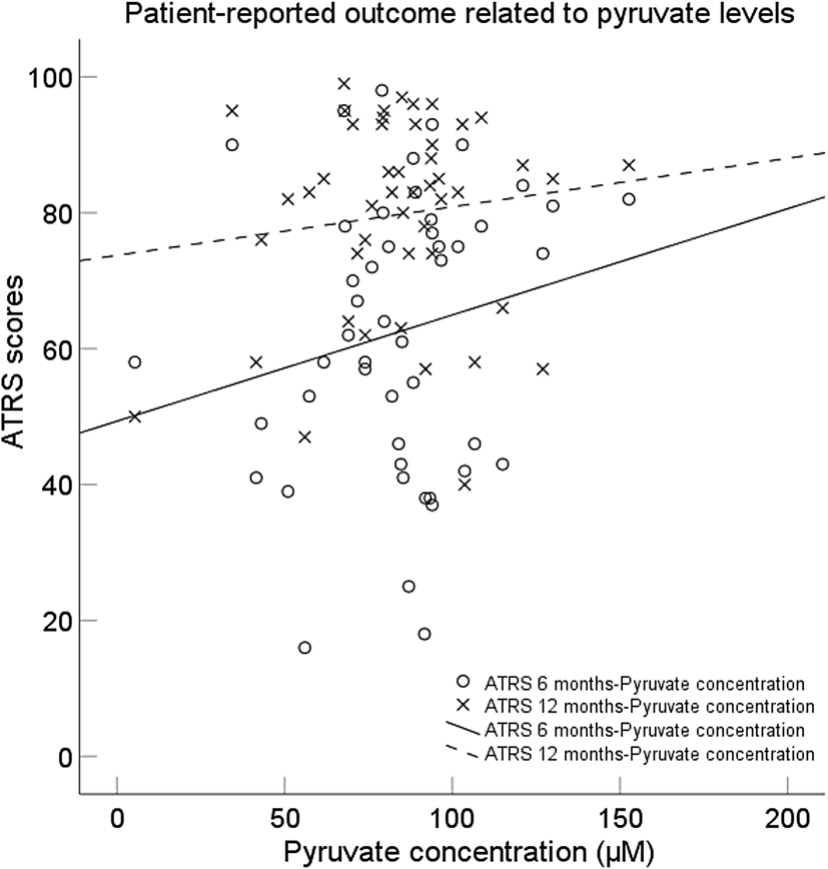
Patient-reported outcome at 6 and 12 months, in relation to pyruvate concentration

To predict ATRS at 6 months, a multiple linear regression model was created based on patient characteristics (age, sex, BMI, mean number of steps taken), and the mean pyruvate and lactate concentrations in the healing tendon. Pyruvate concentration was demonstrated as the single factor associated to ATRS at 6 months (*R*^2^ = 0.32, *p* = 0.003). The model predicted an increase in the score of ATRS at 6 months equal to 36.4 + 0.56 for every 1 µM increase of pyruvate concentration in the healing tendon (Fig. 4, Table 3).

**Table 3:** Patient-reported outcome ATRS at 6 months in relation to pyruvate/lactate

Dependent outcome variable	Mean value (SD)	Multiple linear regression Explanatory variables
ATRS total score	63.2 (20.0)	*F*(1, 24) = 11.03, *p* = 0.003, *R*^2^ = 0.32 Pyruvate concentration
Limited strength in the calf/tendon/foot	5.7 (2.3)	*F*(1, 24) = 9.51, *p* = 0.005, *R*^2^ = 0.28 Pyruvate concentration
Tired in the calf/tendon/foot	6.5 (2.4)	*F*(1, 24) = 7.01, *p* = 0.014, *R*^2^ = 0.23 Pyruvate concentration
Stiffness in the calf/tendon/foot	6.1 (2.5)	n.s
Pain in the calf/tendon/foot	8.1 (2.2)	*F*(1, 24) = 12.74, *p* = 0.002, *R*^2^ = 0.35 Pyruvate concentration
Limited ADL	7.4 (2.4)	*F*(3, 22) = 7.54, *p* ≤ 0.001, *R*^2^ = 0.51 Pyruvate concentration, BMI, Age
Limited on uneven surface	7.4 (2.4)	n.s
Limited in stairs/hills	6.9 (2.6)	n.s
Limited when running	4.7 (2.9)	*F*(1, 24) = 7.19, *p* = 0.013, *R*^2^ = 0.23 Pyruvate concentration
Limited when jumping	4.4 (2.7)	*F*(1, 24) = 6.52, *p* = 0.017, *R*^2^ = 0.21 Pyruvate concentration
Limited in physical work	6.5 (2.6)	*F*(1, 24) = 11.86, *p* = 0.002, *R*^2^ = 0.33 Pyruvate concentration

*n.s*. non significant correlation, *SD *standard deviation

Pyruvate concentration in the healing tendon was in the multiple linear regression demonstrated as the most significant factor which influenced 7 out of 10 subscales of the ATRS at 6 months. For 6 of the ATRS subscales, pyruvate was the single significant factor in the regression model. For limited ADL at 6 months, pyruvate concentration together with age and BMI showed a significant regression’s equation with a positive correlation (*R*^2^ = 0.51, *p* ≤ 0.001). Pyruvate concentration was not significantly associated with limb stiffness, limitations on uneven surface or limitations in stairs (Table 3).

#### Outcome at 12 months

To predict ATRS at 12 months, a multiple linear regression model was created based on patient characteristics (age, sex, BMI, mean number of steps taken), and the mean pyruvate and lactate concentrations in the healing tendon. Pyruvate concentration was demonstrated as the single factor associated to ATRS at 12 months (*R*^2^ = 0.37, *p* = 0.004). The model predicted an increase in the score of ATRS at 12 months equal to 58.6 + 0.61 for every 1 µM increase of pyruvate concentration in the healing tendon (Fig. 4, Table 4).

**Table 4:** Patient-reported outcome ATRS at 12 months in relation to pyruvate/lactate

Dependent outcome variable	Mean value (SD)	Multiple linear regression Explanatory variables
ATRS total score	79.5 (17.5)	*F*(1,18) = 10.73, *p* = 0.004, *R*^2^ = 0.37 Pyruvate concentration
Limited strength in the calf/tendon/foot	7.2 (2.3)	*F*(1, 18) = 4.60, *p* = 0.046, *R*^2^ = 0.20 Lactate concentration
Tired in the calf/tendon/foot	7.8 (2.1)	*F*(1, 18) = 6.88, *p* = 0.017, *R*^2^ = 0.28 Pyruvate concentration
Stiffness in the calf/tendon/foot	7.5 (2.3)	*F*(1, 18) = 4.91, *p* = 0.040, *R*^2^ = 0.21 Pyruvate concentration
Pain in the calf/tendon/foot	9.1 (1.3)	*F*(3,16) = 10.78, *p* ≤ 0.001, *R*^2^ = 0.67 Pyruvate concentration, Podometer mean 1–10, Lactate concentration
Limited ADL	8.8 (1.6)	*F*(1,18) = 9.25, *p* = 0.007, *R*^2^ = 0.34 Pyruvate concentration
Limited on uneven surface	8.9 (1.5)	*F*(2,17) = 5.02,* p* = 0.019, *R*^2^ = 0.37 Pyruvate concentration, Lactate concentration
Limited in stairs/hills	8.3 (1.9)	*F*(1, 18) = 11.56, *p* = 0.003, *R*^2^ = 0.39 Pyruvate concentration
Limited when running	7.3 (2.6)	*F*(1, 18) = 15.63, *p* ≤ 0.001, *R*^2^ = 0.47 Pyruvate concentration
Limited when jumping	6.7 (2.7)	n.s
Limited in physical work	8.3 (2.1)	*F*(1,18) = 7.22, *p* = 0.015, *R*^2^ = 0.29 Pyruvate concentration

*n.s.* non significant correlation, *SD *standard deviation

Pyruvate concentration in the healing tendon was shown to be the single significant determining factor for 6 out of 10 ATRS subscales at 12 months. Limitations on uneven surface was significantly predicted by pyruvate together with lactate concentrations (*R*^2^ = 0.37, *p* = 0.019). Patients experience of pain was significantly related to pyruvate and lactate concentrations together with pedometer data (*R*^2^ = 0.67, *p* ≤ 0.001). Lactate concentration was demonstrated to be the single significant factor correlating to limitations in strength (*R*^2^ = 0.20, *p* = 0.046) (Table 4).

### Adverse events

A total of 147 adverse events were reported (5 re-ruptures, 12 infections, 130 DVT). To investigate the true effect of the metabolites on adverse events, a multiple linear regression was performed based on patient characteristics (age, sex, BMI, mean number of steps taken), and the mean concentrations of pyruvate and lactate in the healing tendon. Mean pyruvate and lactate concentrations were not significantly associated with adverse events after ATR repair.

## Discussion

The most important finding of the present study demonstrates that pyruvate concentrations in the healing Achilles tendon at two weeks after rupture may predict the patient-reported outcome at both 6 and 12-months post-surgery. These findings conclude that efforts should be made to develop methods, mainly improved early mobilization protocols, aimed at increasing pyruvate concentrations after injury to promote tendon healing and thereby improve patient outcome.

A novel observation of this study was that, among several factors investigated, only the number of steps taken during the first post-operative ten days was correlated to increased concentrations of both pyruvate and lactate in the healing tendon. This finding suggests that early mobilization, i.e. increased physical activity, after tendon injury and surgery is essential for upregulation of metabolites involved in tendon healing. The results moreover implicate that patient characteristics, degree of physical activity prior to injury, time to surgery, duration of operative time, and degree of weight-bearing do not significantly affect pyruvate and lactate concentrations in the healing tendon.

The observation that pyruvate concentrations during early tendon healing was related not only to ATRS at 6 months but also to ATRS at 12 months, corroborates the findings and suggests that pyruvate metabolism possesses a vital control of the tendon healing process, which affects the daily life of the patients over a long time.

The conception that pyruvate exerts crucial actions in tendon healing is supported by a recent study [2]. During wound healing, pyruvate has been shown to display anti-fibrotic and antioxidant actions, which reduce the fibrotic collagen type III in favour of the mature and strong collagen type I [10, 22, 31]. During the tendon´s regenerative repair phase, conversion of collagen type III into collagen type I is essential for improving the mechanical properties of the healing Achilles tendon [2]. It may prove that higher pyruvate levels represent prerequisites for optimal energy supply during energy demanding tendon repair phases (Fig. 2).

The observed association between higher concentrations of pyruvate and reduced tiredness, less stiffness and less limitations in activities of daily living, imply that pyruvate have anti-fibrotic and anti-inflammatory properties, thereby improving patient outcome both at 6- and 12 months. This interpretation is supported by a recent study showing that pyruvate injections after injury may reduce oxidative stress and inflammation [22]. The mechanical strength of tendon collagen fibrils is impaired by oxidative stress, suggesting that pyruvate may improve the mechanical properties in tendon healing [7]. Addition of pyruvate after post-ischemic myocardial arrest in pigs lowered the oxidative stress and improved the function of the brain [22]. Supplementation with local sodium pyruvate injections during wound healing has also been demonstrated to accelerate migration of fibroblasts and myoblasts to promote wound gap closure [16].

The exact timing of the conceivable effects of pyruvate on tendon healing is not given from this study. Our assessment of highly elevated pyruvate concentrations at two weeks post-operatively, however, indicate that pyruvate may exert effects in the late inflammatory phase, in the proliferative phase and in the early remodelling phase of tendon healing. This observation is in line with the anti-inflammatory, regenerative and anti-oxidative effects of pyruvate demonstrated by other studies [14, 22, 32]. Moreover, a recent animal study demonstrate that pyruvate production is upregulated the first 4 weeks after Achilles tendon injury [31]. The study furthermore corroborated the essential effects of pyruvate by demonstrating that the inhibition of pyruvate dehydrogenase kinase, which allows for an elevated aerobic metabolism of pyruvate, markedly enhanced the mechanical properties of the healing Achilles tendon and also improved the alignment of the collagen fibres [20].

The observation that improved patient outcome is associated with higher concentrations of pyruvate, is supported by studies demonstrating the underlying mechanisms of action of pyruvate in tendon healing and indicative of a potential tendon regenerative effect of pyruvate supplementation. The idea of adding pyruvate to the healing tendon to promote the regenerative repair process, is appealing and substantiated by other studies, but necessitates verification from prospective studies [2, 16, 31].

The second main finding from this study pertain to lactate exhibiting less associations with the patient-reported outcome measures compared to pyruvate. However, since pyruvate is reduced to lactate in anaerobic environments, their metabolism is interdependent, and lactate serves as a marker for low oxygen conditions [32]. The observation at two weeks post-injury that lactate concentrations was upregulated in the healing tendon compared to the contralateral uninjured tendon, suggests that lactate metabolism is active and that some parts of the healing process at this time-point may be anaerobic.

The observation that higher concentrations of lactate and pyruvate together were correlated with less experience of pain and less limitations on uneven surface at one year demonstrate a positive interaction between these two metabolites. The observation that lactate exerted a positive effect on healing is strengthened by earlier research showing that lactate promotes collagen production by upregulating the expression of transforming growth factor beta and its receptor [30].

The earlier results showing that lactate promotes collagen production, would also be well in line with our finding demonstrating increased lactate concentrations as the single factor related to less strength limitations in the injured limb. An earlier study that investigated the ratio of both lactate and pyruvate between the injured- and uninjured limb supports the findings that the lactate/pyruvate metabolism is important for tendon healing also when it comes to functional outcome [2].

The secondary aim of this study was additionally to examine which underlying factors that are involved in the regulation of pyruvate- and lactate metabolism. The finding that the number of steps taken are positively correlated to the concentrations of pyruvate and lactate in the healing tendon, suggests a previously unknown mechanism underlying the positive effects of early mobilization in Achilles tendon healing. The observation furthermore indicates that supplementation of pyruvate and/or lactate to the healing tendon may be a viable treatment in case of poor mobility or other reasons for low endogenous pyruvate or lactate concentrations. These hypotheses, however, warrant further investigations.

A potential limitation of this study is the retrospective nature. Multiple surgeons may be a possible source of bias, but all surgeons used the same standardized operative techniques according to a predefined study protocol. Different postoperative treatment protocols could potentially influence the concentrations of metabolites in the healing tendon, although our correlation analyses showed no influence. A similar investigation has looked at the ratio of pyruvate and lactate concentrations in tendon healing, however, such ratios may introduce biases in interpretation [2]. Therefore, this study with more than 100 patients undergoing microdialysis with subsequent establishment of the mean concentrations of pyruvate and lactate, present one of the largest studies on peritendinous microdialysis ever performed. A drawback of this study was, however, that not all patients that performed the microdialysis were assessed with pedometer. However, meticulous examination of underlying factors affecting pyruvate- and lactate concentrations, as well as analyses taking careful consideration of confounding factors, were undertaken. Further studies should investigate the influence of different treatment methods on the concentrations of pyruvate and lactate in tendon healing. In clinical practice efforts should be made to utilize early mobilization protocols with high step counts, aimed at increasing pyruvate concentrations after injury to promote tendon healing and thereby improve patient outcome [4, 28].

## Conclusions

Patients with ATR exhibit, at both 6- and 12-months, improved patient-reported outcome related to higher concentrations of pyruvate in the healing Achilles tendon. The glycolysis metabolite’s lactate, which is formed under anaerobic conditions, exhibits less relationship to patient outcome after ATR. Early mobilization with higher number of steps taken is a clinical key to increase the concentrations of both pyruvate and lactate and to improve the patient-reported outcome.
